# Roles of Steroids in Preventing Esophageal Stricture after Endoscopic Resection

**DOI:** 10.1155/2019/5380815

**Published:** 2019-04-01

**Authors:** Yu Qiu, Ruihua Shi

**Affiliations:** ^1^Medical School of Southeast University, No. 87 Ding Jia Qiao, Nanjing, Jiangsu 210009, China; ^2^Department of Gastroenterology, Zhongda Hospital, Medical School of Southeast University, No. 87 Ding Jia Qiao, Nanjing, Jiangsu 210009, China

## Abstract

**Background and Purposes:**

Endoscopic resection has been worldwide recognized as a treatment strategy for early esophageal lesions. The occurrence of esophageal stricture after endoscopic resection will reduce the quality of life of patients. This study will evaluate the efficacy and safety of steroids in the prevention of esophageal stricture after endoscopic resection and the influence of different steroid administration methods.

**Methods:**

In the relevant literature database, literature from 2008 to 2018 is retrieved by using preset keywords, the search results are carefully screened, and the conclusion of the literature is synthesized to form arguments and draw conclusions.

**Results:**

73 articles met our requirements. Oral steroid administration, not prophylactic endoscopic balloon dilation alone, was effective in preventing esophagostenosis after esophagoscopic treatment and reducing the number of repeated endoscopic balloon dilations even after extensive endoscopic resection. Local steroid injection is useful and economy for preventing esophageal stricture, even though it may raise the risk of perforation during dilations. A wider range of circumferential mucosal defects is an independent predictor for stricture formation for patents given preventive steroid injections after endoscopic submucosal dissection. For complete circular mucosal defect, the further researches are essential to investigate the role of local steroid injection. The effect of other methods such as steroid gel, intravenous infusion of steroid, and novel steroid filling methods require more confirmation.

**Conclusions:**

Therefore, steroids play an irreplaceable role in preventing esophageal stricture after endoscopic resection. Oral and local injections of steroids are the two most acceptable methods and more prospective studies are needed to compare the effectiveness and safety of these two methods.

## 1. Introduction

Endoscopic resection has been worldwide recognized as a proper strategy for superficial esophageal dysplasia and carcinoma due to its feature of minimal invasion. As an alternative to esophagectomy, endoscopic mucosal resection (EMR) had been originally applied in the treatment for localized neoplasm, because of the better quality of life after surgery. Segmental circumcision of esophageal intima can successfully remove most of the stenosis lesions [1, 2]. However, the operation of segmental circumcision is easy to cause the relapse of superficial esophageal cancer (SEC) [3, 4]. In the past few years, focus of EMR has been gradually replaced by endoscopic submucosal dissection (ESD) for SEC. ESD allows the entire resection of the lesion regardless of its size and has a lower recurrence rate compared to EMR [5].

The incidence of esophageal stricture after endoscopic mucosal resection is rather high; patients often have to undergo radiofrequency ablation again to eliminate proliferative mucosa [6]. The definition of esophageal stenosis is a narrowing of the esophageal lumen found on endoscopy, which cannot be passed by a standard endoscope or is related to dysphagia [7]. The stricture rates after EMR are 1.3%-4.9% and 3%-11.6% for ESD [8, 9], but the comparability of these rates is low, because the circular extent of the mucosal defect differs among the studies. Chikatoshi et al.'s research found that extensive defect of esophageal mucosa is one of the important predictors of esophageal stricture after ESD [10], which has been verified by many other authors [11, 12]. In addition, Satoshi et al. added that T1A m2 histological depth was another important factor related to post-ESD esophageal stenosis [13].

The process of esophageal healing and stricture formation after endoscopic resection involves three stages: the first stage is an injury of epithelium, resulting in the damage of the barrier of the epithelial and making the submucosal layers exposed to food boluses, acid, or bile reflux and esophageal fungal or bacterial flora. The second stage is the activation of immune system [14] which is characterised by the hyperplasia of granulation tissue, including inflammatory cell infiltration and angiogenesis [15]. The third stage is scar tissue formation, which involves fibroblastic and myofibroblastic proliferation with the stimulation of cytokines like TNF-a, TGF-b1, IL-6, IL-1, IL-17A, PDGF, and so on [16].

Most post-ESD strictures are refractory and repeated endoscopic dilations are required [17]. Endoscopic dilation is effective for stricture of internal diameter after endoscopic esophageal mucosal dissection, but there is a high rate of recurrence [18]. Besides, repeated endoscopic balloon dilations (EBDs) would give rise to complications including perforation and bleeding [19]. It is reported that 7.1-9% of the patients suffer from perforation after repeated endoscopic dilation [20]. In recent years, there are many treatments for esophageal stricture after esophageal mucosal exfoliation, such as stents placement, botulinum toxin injection, oral tranilast, and local autologous cell transplantation. Numerous studies have shown good therapeutic effects, while more larger multicenters investigations are required. Therefore, prevention of esophageal stenosis after endoscopic resection is necessary for endoscopic therapy to develop.

Due to the double function of anti-inflammation, treatment of esophageal stricture by oral or local steroid injection has become the preferred option [21]. This review aims to evaluate the efficacy and safety of steroids in the prevention of esophageal stricture after endoscopic resection and evaluate the efficacy of steroids in different routes of administration.

## 2. Methods

We searched the relevant literature on PubMed and Web of Science (from 2008 to December 2018) that focused on steroid therapy for the prevention of esophageal stenosis after extensive endoscopic resections. The search terms included “esophageal stenosis or stricture” and “steroid” and animal studies were excluded from all literature. In addition, prevention was used as the criterion for research purposes, and treatment goals were excluded. As is seen from references, 73 articles eventually met our requirements.

### 2.1. Oral Steroid Method

Naoyuki et al. conducted a large number of clinical trials, which proved for the first time that oral steroids have a good therapeutic effect on esophageal stricture after esophageal mucosal stripping [22]. In the study, patients were given prednisolone 30 mg daily from the third day after ESD, and the dosage of prednisolone decreased by 5 mg per week until the end of the eighth week of the experiment. This study included 41 patients treated with ESD, whose circumference was more than three-quarters. The incidence of esophageal stricture in oral prednisolone group (5.3%) was significantly lower than that in pre-EBD group (31.8%, P=0.03). The average number of times requiring EBD treatment was 1.7 in the oral prednisolone group and 15.6 in the pre-EBD group (P < 0.0001). Later, Mikinori et al. [23] and Hiroki et al. [24] investigated oral steroid for prevention of esophageal stenosis by applying the Yamaguchi protocol with little change. It is worth mentioning that Hiroki et al. [24] paid attention to complete circumferential ESD, which showed that stenosis after ESD occurred in all patients. However, compared with EBD alone, the number of patients requiring EBD after steroid therapy was significantly reduced (13.8 versus 33.5, P < 0.001) and shorter than the period of physical rehabilitation (4.8 versus 14.2 months, P<0.005). Besides, some other investigations stated that oral corticosteroids can prevent esophageal stenosis after esophageal ESD [25–29]. Recently, in a survey conducted by Iizuka et al., 22 post-ESD patients requiring EBD treatment were included [25]. They investigated a modified oral steroid administration: in the first three weeks, 30 mg prednisolone was given orally every day, and the dosage of prednisolone was reduced by 5 mg every three weeks. The results showed that the esophageal stricture rate (36.4%) in the modified group was significantly lower than that in the original group (82%, P=0.04) after ESD compared with the traditional 8-week decreasing regimen. In the improved group, the number of patients requiring EBD treatment also decreased significantly (6.2 versus 19.4, P=0.023). Therefore, compared with previous methods, this improved administration is exciting in preventing esophageal stricture after endoscopic surgery. 

Current studies have fully confirmed the preventive and therapeutic effects of steroids on esophageal stricture after esophageal endometrectomy, but more studies are needed to determine the safest and most effective way of administration, dosage, and time of administration. As we can see from Table 1, most investigations adopt 8-week therapy despite the differentiation of dose and interval. However, the total dose of prednisolone usually exceeds 1,000 mg in eight weeks and it has been reported that patients undergoing systemic steroid administration for 21 days or taking more than 700 mg prednisolone have a raised risk of infection [30]. Oliveira et al. confirmed that the use of high dose glucocorticoids would lead to serious complications such as gastrointestinal ulcer, elevated blood sugar, immunosuppression, osteoporosis, and even systemic infection [31]. For example, pneumocystis pneumonia (PCP) is caused by systemic immune suppression in some patients who take high doses of steroids for a long time. The mortality rate of PCP is very high [32]. Limper et al. suggested that steroids administration in doses greater than 20 mg for 1 month or longer should call for prophylaxis for PCP [33]. In addition, Tsukasa et al. reported an 85-year-old man who had almost completely resected the periphery of esophageal mucosa and used steroids to prevent esophageal stricture after surgery [34]. After six weeks' steroid treatment with a total of 1120 mg prednisolone, he developed high fever and finally was diagnosed with infection of nocardiosis. These cases suggest the importance and necessity of preventing fatal infections in patients receiving systemic steroid treatment after endoscopic resection and it is necessary to reduce the amount and shorten the duration of systemic steroid therapy. Stuck et al. pointed out that the early application of steroids after esophageal mucosal resection is helpful to cut off the inflammation process as early as possible, and patients will get more benefits [35]. Hiroki et al.'s study compared the occurrence and progression of esophageal stricture after early and late steroid therapy for ESD [24]. In their study, three patients in EBD therapy alone group finally orally intake prednisolone after about 158 days due to the fact that they failed to responded to EBD therapy alone. Therefore, they added a subgroup analysis to compare these three additional patients with 10 original patients in the steroid plus EBD group. The results showed that the time needed for EBD in steroid + EBD group was significantly reduced (13.8 versus 46.0, P < 0.002), and the total EBD time was significantly shortened (4.8 versus 17.5, P<0.005). 3-7 days after injury is the key period for collagen deposition and fibrosis, which may be the main mechanism for the early benefit of steroid hormone application [36]. More studies with larger sample size comparing early and late oral steroid therapy are needed to confirm and further explain the results of the study and explore the mechanism of steroids exerting local anti-inflammatory and antifibrotic effects. To conclude, early oral steroid administration, with proper amount and duration, not preventive EBD alone, was effective in preventing esophageal stricture and avoiding EBD. Further larger scale of investigations are required before this therapeutic option can be widespread.

### 2.2. Local Steroid Injection

Holder et al. [37, 38] took the lead in studying the effect of corticosteroid local injection therapy on benign esophageal stricture in dogs and children. Since then, its clinical application has gradually increased [39–41]. In recent years, local steroid injection therapy has been widely used to prevent the formation of stenosis after SEC [7, 42]. Kouichi et al. had explored the healing process of local ulcers after ESD by injecting steroids into pigs' esophagus [43]. After Satoru's study, they used triamcinolone acetonide at doses and injection intervals [7]. What the conclusion they drew was that local steroid injection seemed to be effective to prevent the stricture after esophageal ESD, but as for the optimal injection technique, frequency, and dose of triamcinolone, it requires further people-based studies. As is shown in Table 2, several prospective or retrospective studies have manifested that local steroid injection at the ulcer base significantly reduced esophageal stricture rate after endoscopic resection.

Satoru et al. [7] conducted the first study to manifest that endoscopic triamcinolone injection (ETI) leads to decreased stricture rate and numbers of EBD in patients undergoing ESD for SEC in 2011. Based on the knowledge that fibroblasts begin to proliferate 3 to 7 days after local trauma[36], they began ETI at this time point and injected steroid hormones into the site of ulcer. The results showed that when triamcinolone acetonide was injected into the shallow layer with equal interval of 2 mg, the total dose of triamcinolone acetonide was 18-62 mg; there were no significant complications such as delayed perforation and local abscess. Then Hanaoka et al. [44] conducted a prospective study on 30 patients with esophageal squamous cell carcinoma (ESCC) treated with EDS which had a periesophageal defect of more than 3/4, but not a whole-week defect. Compared with 29 patients without ETI treatment, the rate of esophageal stricture in the experimental group was significantly reduced (10% versus 66%, P<0.0001), and the number of patients requiring EBD treatment in the later stage was significantly reduced (0-2 versus 0-15, P<0.0001). One patient in study group suffered from a submucosal tear and another suffered from bleeding, which were both not direct results of EBD. In addition, for patients with whole circumferential mucosal defects after esophageal ESD, Takahashi et al. [45] conducted a randomized controlled trial and they found that ETI did not reduce the stricture rate, but decreased the mean times of dilatation sessions (from 12.5 to 6.1), indicating that, in some patients, local steroid injection prevented the rapid healing of esophageal ulcer. Similarly, the results of a single-center randomized controlled trial conducted by Mei-Dong Xu et al. [46] showed that local steroid hormone injection was effective in preventing stenosis in patients with peripheral esophageal defects less than half a week after ESD. Yasuaki et al.'s [47] propensity score matching analysis excluded steroid injection selection bias and other confusing factors. They used two LSI methods: one was to inject 80 mg triamcinolone acetonide (TA) immediately after ESD; the other was to inject 6.6 mg dexamethasone and Twi immediately after ESD. Stenosis was found in 1 of 12 lesions treated with TA (8.3%) and in 2 of 16 lesions treated with dexamethasone (12.5%) (p = 1.00). Their results also indicated that LSI is usefully limited to mucosal defects less than 3/4 of their esophageal circumference. Therefore, what on earth are the risk factors for stricture formation in patients who received ETI after esophageal ESD? Recently, a study by Yasuaki et al. [48] assessed the risk factors for preventing esophageal stricture by routine prophylactic steroid injection after ESD. They concluded that a wide mucosal defect (odds ratio: 2.42; 95% confidence interval: 1.01-5.80; P=0.048) was an independent predictor of stenosis. The cut-off value related to stenosis formation was 5/6 of the peripheral mucosal defect. Tendency analysis showed that the rate of esophageal stricture was increased in patients with circumference greater than 5/6, compared with those with less than 5/6 mucosal defects (odds ratio: 5.70; 95% confidence interval: 1. 61-20.18; P= 0.007).

Yoshiki et al. [49] stated that the boundary between esophageal submucosa and muscular layer was blurred and the muscular layer was partially ruptured after local steroid injection. Thereafter, Yoshiki's team conducted a central retrospective study to assess the prognosis of patients with periesophageal mucosal defects more than three-quarters of the time after ESD. Their findings found that 43% of LSI patients had esophageal stricture, compared with 90% of nonpreventive patients, and only esophageal perforation occurred in LSI patients [50]. Yamashina et al. [51] reported the cases of delayed perforation caused by tissue damage caused by LSI. They believed that injection needles should be avoided to puncture the intrinsic muscles directly. Therefore, mild puncture of the residual submucosa should be performed without needling deeper. EBD should also be performed cautiously. Regarding the amount of steroids injected, studies from Yasuaki [52] showed that a single dose of 80 mg TA had sufficient protection against stenosis, although this dose was lower than previous studies. Finally, according to the statistical data of Furuhashi et al. [53], each case in LSI group used 60±28 mg of triamcinolone acetonide and the Japanese public insurance system proposed a reduction of $9330 in medical expenses. In conclusion, LSI is useful and economical in preventing esophageal stricture after ESD, although it may increase the risk of perforation during EBD. A wide mucosal defect was an independent predictor of stenosis after LSI and the cut-off value was 5/6 of the peripheral mucosal defect. For complete circular ESD, more prospective studies are needed to investigate the role of LSI.

### 2.3. Other Methods Using Steroids

In addition to oral intake and local injection, steroids can also be applied in other forms such as sterid gel, intravenous drip infusion, and TA filling method to prevent esophageal stricture after ESD. In 2013, Hirohito Mori et al. [54] reported an innovative method using combined steroid gel application and balloon dilatation to prevent esophageal strictures after ESD, compared with steroid injection. They drew a conclusion that steroid gel application is more effective than local injection in terms of prevention of esophageal stricture after ESD. However, the results of Hirohito Mori's study which was questioned by Yang Fan et al. [55] have made great contributions in optimizing and expanding the design process, data analysis, and results study of the experiment in this direction. In addition, Satoshi et al. [56] reported intravenous steroid infusion for the first time in 2010 as a case report. A 61-year-old male patient with SEC underwent esophageal ESD examination and treatment. Multiple myeloma and multiple bone metastases were found during further treatment. Therefore, from the 9th day after ESD, 40 mg dexamethasone was orally administered daily for three consecutive days, a total of three courses of treatment. Subsequently, the patient received peripheral blood stem cell transplantation, which was successful. During follow-up, no significant esophageal stricture or typical symptoms were found in this patient. This study opens a new era of pulsed hormone therapy. In 2015, Nakamura et al. [57] prospectively studied the efficacy and safety of steroid pulse therapy in preventing esophageal stricture after ESD. Esophageal cancer was diagnosed. No serious life-threatening complications related to steroid pulse therapy have been found. The median interval between ESD and EBD was 18 days (15-21 days) and 2 days (1-6 days). Although the occurrence of esophageal stricture seems inevitable, pulsed steroid regimens can significantly reduce the frequency and overall time of EBD. Similarly, Nakamura et al. [58, 59] conducted a prospective study of 11 patients in 2017 and found that pulsed steroid regimens were extremely safe. Unfortunately, no preventive effect on esophageal stricture was found. On this basis, they proposed a local esophageal tamponade therapy using triamcinolone acetonide (TA), which is to inject TA saline solution into the esophagus for a period of time, expecting the drug to penetrate evenly into the broader resected surface. The results showed that esophageal stricture was effectively controlled in all patients. Unfortunately, the results of large-scale data analysis are still lacking in this study.

## 3. Discussion

Wound healing is a process involving inflammation, hyperplasia, and remodeling, and scar formation is considered to be a part of wound healing. Collagen is the major fibrous connective tissue protein in scars [40]. In theory, steroids are the most suitable treatment agent for scar because they can inhibit inflammation response, collagen synthesis and fibroblast proliferation [60]. At present, oral or local steroid injection has become a common clinical treatment to prevent esophageal stricture after ESD. Compared with EBD, the treatment results are more economical and effective, and patients suffer less pain [51]. However, there are high-risk complications such as delayed ulcer healing, ulcer, or perforation caused by injection injury in LSI treatment. The oral steroid hormone pathway is not 100% safe. Severe complications such as immunosuppression, pulmonary infection, elevated blood sugar, osteoporosis, and mental disorders often occur. Tsukasa et al. reported a case of severe disseminated nocardiosis during oral steroid therapy [34]. Wang et al. made a meta-analysis with the objectives to evaluate the efficacy of steroids in preventing esophageal stricture after ESD and to conclude that LSI is superior to oral steroids in reducing EBD [61]. Recently, Yang et al. conducted another network meta-analysis to assess the efficacy and safety of different steroid applications in the prevention of esophageal stricture after endoscopic submucosal dissection [62]. Interestingly, they drew the conclusion that long-term oral steroid (at least 12-week period with more than 1470-mg prednisolone) might be the superior prevention for postoperative stricture with satisfying efficacy, comparing with preemptive EBD, median-term oral steroid, short-term oral steroid, and steroid injection therapy. However, it has been reported that patients undergoing systemic steroid administration for 21 days or taking more than 700 mg prednisolone have a raised risk of infection [30]. Therefore, more prospective comparative studies are needed to clarify the effectiveness and safety of oral steroid and local injection methods.

Multicenter prospective randomized controlled trials (JCOG1217) are currently comparing the efficacy of prophylactic oral steroids and LSI in the treatment of noncircular lesions [63]. Besides, Kawaguchi et al. [64] proposed a combined therapy called “sequential steroid therapy”, which means LSI of triamcinolone on the day of ESD followed by oral intake of prednisolone for the next few days. The effectiveness of this novel therapy requires confirmation by more prospective studies. According to a retrospective study conducted by Kadota et al. [65], stricture rates were assessed based on different widths of mucosal defects caused by ESD. Their findings confirm that prophylactic steroid use is effective in the treatment of esophageal stricture in patients with mucosal defect 7/8 weeks or more after ESD, but ineffective after full-week mucosal defect. Further investigations with larger sample size are required. In addition, more and more researchers take steroid therapy, either oral steroids or LSI, as a basic therapy for preventing esophageal stricture after ESD, combining with some other methods like stents insertion and polyglycolic acid sheets, which may have additive or synergistic effects. Nowadays, new methods for prevention and treatment of esophageal stricture after ESD have been widely studied, such as esophageal stent implantation, polyglycolic acid shielding, autologous oral epithelial cell transplantation, and so on. Many new methods have good results and broad prospects for clinical application, but large-scale studies are still needed to obtain more clinical data.

## 4. Conclusions

Steroids play an irreplaceable role in preventing stenosis after esophageal ESD. Its usage mainly includes oral steroid method, local steroid injection method, other methods such as steroid gel, intravenous infusion of steroid, and novel steroid filling methods. Currently the most widely accepted methods are oral steroid and LSI, and both of them have advantages and disadvantages; more researches are needed to compare the effectiveness and safety of these two methods. Early oral steroid administration, with proper amount and duration, not preventive EBD alone, was effective in preventing esophageal stricture and avoiding EBD. LSI is useful and economical in preventing esophageal stricture after ESD. A wide mucosal defect was an independent predictor of stenosis after LSI and the cut-off value was 5/6 of the peripheral mucosal defect. For complete circular ESD, more prospective studies are needed to investigate the role of LSI. Besides, a combination of these two methods, called sequential therapy, is a novel therapy requiring confirmation by more prospective studies. Last but not least, steroid therapy combining with other treatments like stents insertion and polyglycolic acid sheets may have additive or synergistic effects on the prevention of esophageal stricture after ESD.

## Figures and Tables

**Table 1:** Clinical outcomes of oral steroid method of preventing esophageal stricture after endoscopic resection.

Author, year	Design	Setting	Sample size (treated/control)	Follow-up	Stricture rate	Mean or median number of EBD	Adverse event
Lizuka, 2018 [25]	Retrospective cohort study	ESD 100% SEC	11/11	Gastroscopy: 1, 4, 8, 12, 16, and 20 weeks after ESD	36.4%VS 82% (P=0.04)	6.2 VS 19.4 (P=0.023)	8 VS 3 (P=0.043)
Zhou, 2017 [26]	Retrospective cohort study	ESD >75% ESCC	13/10	Gastroscopy: 1, 3, 6, and 12 months after ESD	23.1%VS 80% (P=0.007)	0.69 VS 13.5 (P=0.004)	None
Jean-Philippe,2017 [27]	Single-center retrospective study	EMR >50% Barrett's esophagus	29/0	Gastroscopy: 2, 6, 12, and 24 months after EMR	13%	2	None
Mikinori, 2015[23]	Retrospective cohort study	ESD >75% ESCC	17/16	Gastroscopy: 8 weeks after ESD	17. 6%VS 68.7% P<0.01	4.6 VS 8.1 P<0.01	None
Hiroki, 2013 [24]	Retrospective cohort study	ESD 100% SEC	10/13	Gastroscopy: 7 days, 2-, 3-, and 4-week interval until 9^th^week after negative endoscopy	Both 100%	13.8 VS 33.5 P<0.001	None
Naoyuki, 2011 [28]	Case report	ESD 100% ESCC	1/0	Gastroscopy: 1, 3, and 6 months after ESD	0%	0	None
Hajime, 2011 [29]	Retrospective study	ESD 100% ESCC	4/3	Gastroscopy: 3, 6, and 12 months after ESD and then annually thereafter	Both 100%	3.25 VS 32.67 (P<0.05)	None
Naoyuki, 2011 [22]	Retrospective study	ESD >75% ESCC	19/22	Gastroscopy: 1, 3, 6, and 12 months after ESD and then annually thereafter	5.3%VS31.8% (P=0. 03)	1.7 VS 15.6 (P<0. 0001)	None

SEC, superficial esophageal cancer; ESCC, esophageal squamous cell carcinomas.

**Table 2:** Clinical outcomes of local steroid injection of preventing esophageal stricture after endoscopic resection.

Author, year	Design	Setting	Sample size (treated/control)	Follow-up	Stricture rate	Mean or median number of EBD	Adverse event
Tsujii, 2017 [50]	Retrospective study	ESD>75% SENs	28/10	Gastroscopy: a few months after ESD	43% VS 90% (P=0.012)	8 VS 5 (P>0.05)	5 VS 1
Yasuaki, 2017 [52]	Retrospective study	ESD>2/3but <100% SENs	37/37	Gastroscopy: every 4 weeks after ESD or receiving complaints of dysphagia	18.9% VS 45.9% (P=0. 016)	0.6±1.5 VS 2.8±4.6 (P<0.01)	None
Furuhashi, 2017 [53]	Historical control study	ESD≥30mm diameter or ≥3/4	43/25	Not mentioned	Multivariate analysis: OR:0.041,95%CI:0.007 to 0.24, p<0.001	2.3 VS 10.9	None
Xu, 2016 [46]	Randomized controlled trial	ESD≥1/2 SENs	17/23	Not mentioned	29.4% VS 69.6% (P=0.02)	0.5 VS 1.3 (P=0.16)	1 VS 0
Yoshiki, 2016 [49]	Retrospective study	ESD>1/2 SENs	30/15	Not mentioned	40% VS 60% (P>0.05)	Not significantly different	6 VS 0
Yasuaki, 2016 [47]	Retrospective study	>2/3but<3/4 ESCC	28/28	Gastroscopy: 4 and 8 weeks after the treatment.	10.7% VS 35.7% (P=0.035)	Not mentioned	Not mentioned
Takahashi, 2015 [45]	Prospective study	ESD>75% ESCC	16/16	Not mentioned	62.5% VS 87.5% (P=0. 22)	6.1 VS 12.5 (P=0.04)	1.0% VS 0. 5%
Lee, 2013 [66]	Case report	ESD 100% ESCC	1/0	Gastroscopy: 2 and 4 weeks and 4 months after ESD. Total follow-up time lasts for 6 months.	0%	0	None
Hanaoka, 2012 [44]	Prospective study	ESD>75% but<100% ESCC	30/29	Gastroscopy: whenever patients reported dysphagia and 2 months after ESD in patients without dysphagia.	10% VS 66% (P<0.0001)	0 VS 2 (P<0.0001)	2 VS 0
Satoru,2011 [7]	Retrospective	ESD >75% ESCC	21/20	Gastroscopy: assess for stenosis at 1 week, 1 month, 6 months, and 1 year after ETI.	19% VS 75% (P<0.001)	1.7 VS 6.6 (P<0.001)	None

SENs, superficial esophageal neoplasms; LSI, local steroid injection; DSP, dexamethasone sodium phosphate; TA, triamcinolone acetonide; TTI, topical triamcinolone injection; ESCC, esophageal squamous cell carcinoma.
